# An immune challenge affects growth dynamics, oxidative stress and survival in wild spotless starling nestlings

**DOI:** 10.1242/jeb.250556

**Published:** 2025-08-04

**Authors:** Daniel Parejo-Pulido, Silvia Casquero, Ana Á. Romero-Haro, Lorenzo Pérez-Rodríguez

**Affiliations:** ^1^ Instituto de Investigación en Recursos Cinegéticos (IREC), CSIC-UCLM-JCCM, Ronda de Toledo 12, 13005 Ciudad Real, Spain; ^2^ Departamento de Ecología Evolutiva, Museo Nacional de Ciencias Naturales (MNCN), CSIC, José Gutiérrez Abascal 2, E-28006 Madrid, Spain

**Keywords:** Acute-phase response, Altricial, Compensatory growth, Developmental plasticity, Early-life development, Second wave of clutches

## Abstract

The activation of the immune system in response to a pathogen infection can impose significant costs on growth and oxidative stress. Developing individuals are particularly vulnerable to this challenge, as their physiological systems are still maturing and their rapid growth to achieve an optimal size is resource demanding. Here, we investigated the costs in terms of growth and oxidative stress of an experimentally induced immune challenge in wild spotless starling (*Sturnus unicolor*) nestlings. To this aim, individuals were injected with lipopolysaccharide (LPS), an antigen that triggers an immune response, or a control substance (PBS) in a within-brood design. Although the immune activation impaired the rate of body mass gain within the first 6 h after the challenge, nestlings subsequently showed an accelerated increase in mass within the following 18 h, reaching a similar body mass to the controls by the next day. This represents a compensation (‘catch-up growth’) occurring within as little as 6–24 h after the challenge. Despite this compensation, initially smaller challenged nestlings showed reduced body mass and survival rates by 8 days after treatment compared with smaller control nestlings. The induced immune challenge also led to increased plasma levels of oxidative damage (reactive oxygen metabolites) and antioxidant capacity, supporting the oxidative cost of immune system activation. These findings highlight the costs of mounting an immune response during early life, characterized by an altered growth dynamic and increased oxidative damage, and the condition dependence of these effects.

## Teaser

**Summary:** An immune challenge in wild starling nestlings affects growth trajectories and increases oxidative damage, with lasting effects on smaller chicks’ survival, highlighting the costs of immune activation during early development.

## INTRODUCTION

Conditions experienced during early-life development play a crucial role in shaping an organism's future life path, as they may have profound effects on individual morphology, physiology, breeding success, cognition and behaviour (Cam and Aubry, 2011; Lindström, 1999; Metcalfe and Monaghan, 2001). Nutritional deficits, competition for resources or infectious diseases at this stage can disrupt optimal development, leading to negative consequences in both the short and long term (Cam and Aubry, 2011; Møller et al., 2009; Nettle et al., 2015). To mitigate the detrimental effect of pathogens, organisms have evolved complex immune defences in response to the threat of diseases (Zuk and Stoehr, 2002). However, while the activation of the immune system provides undeniable benefits, it may also impose collateral costs due to trade-offs associated with other fitness-related functions (Eraud et al., 2009; Hasselquist and Nilsson, 2012; Sheldon and Verhulst, 1996). Despite extensive evidence on the costs of immune responses, the mechanisms behind these costs remain poorly understood.

In vertebrates, the activation of the immune response begins with the acute-phase response, a complex, coordinated sequence of events that involves, among other physiological changes, the synthesis and proliferation of leukocytes and acute-phase proteins (e.g. haptoglobins), along with fever and loss of appetite (Hart, 1988; Klasing, 2004; Owen-Ashley and Wingfield, 2007). The processes involved are highly resource demanding, and these resources are typically limited and shared between competing physiological functions. As a result, the organism must redirect them from other non-essential functions, such as skeletal development and growth, to support the immune system and meet the resource demands of fighting the infection (Klasing, 2004; Lochmiller and Deerenberg, 2000). In this sense, numerous studies have shown that immune activation negatively affects growth (e.g. Armour et al., 2020; Bonneaud et al., 2003; Burness et al., 2010; Merino et al., 2000; Soler et al., 2003; Schneeberger et al., 2013; but see Hõrak et al., 2000; Serra et al., 2012; Whitaker and Fair, 2002, for no effects of immune response on growth), either by reducing food intake due to the anorexia induced by the acute-phase response or by diverting resources away from growth to sustain the immune response. In immature organisms such as early-life phases of altricial species, whose survival and future reproductive success depend on achieving an optimal mass and size during this critical and short developmental period (Magrath, 1991; Redondo et al., 2022), the negative impact of mounting an immune response on growth is even stronger. In response, young individuals may engage in compensatory strategies to rapidly recover mass loss and achieve the target optimal body mass, such as ‘catch-up growth’ (Hector and Nakagawa, 2012; Metcalfe and Monaghan, 2001, 2003). However, the path to reach this optimal mass may follow multiple developmental trajectories (Metcalfe, 2024). Although the end-point phenotype may appear similar, these adjusted trajectories may involve concomitant costs, which may vary depending on the specific path taken. These costs include oxidative damage from the increased production of reactive oxygen species during growth or accelerated cellular ageing, which may ultimately decrease survival prospects (Metcalfe and Monaghan, 2001; Stier et al., 2014, 2015).

Additionally, the activation of the immune response itself may directly impair other physiological functions, such as maintenance of oxidative balance (Costantini, 2022; Costantini and Møller, 2009; Sorci and Faivre, 2008). The activation of inflammatory cells involves the release of cytotoxic compounds – including reactive oxygen and nitrogen species – that help destroy pathogens. However, when the antioxidant defences of the organism cannot buffer the over-production of these pro-oxidants, a situation of oxidative stress may arise, leading to damage to host DNA, lipids and proteins, impairing cell functionality (Halliwell and Gutteridge, 2007). Oxidative stress is a key constraint on the expression of fitness-related traits and is associated with accelerated cellular senescence (Costantini, 2024a). Importantly, the antioxidant machinery of the organism is thought to be still under development during early life, making individuals particularly vulnerable to the excess of pro-oxidants (Metcalfe and Alonso-Álvarez, 2010, and references therein). Indeed, it has been shown that the pervasive impact of high levels of oxidative stress during development may have long-lasting consequences on fitness and offspring phenotype (Costantini, 2024b), affecting recruitment probability, reproductive output and breeding capacity at adulthood (e.g. Alonso-Álvarez et al., 2006; Noguera et al., 2011; Romero-Haro and Alonso-Álvarez, 2020).

During the past decades a large body of empirical studies and meta-analyses have addressed the impact of the immune response on body condition and oxidative stress (Costantini, 2022; Costantini and Møller, 2009; Hasselquist and Nilsson, 2012; van der Most et al., 2011). However, the focus of most of these studies is biased towards adult captive individuals, while studies on early life stages or under natural conditions are scarcer (Costantini, 2022; Costantini and Møller, 2009; Hasselquist and Nilsson, 2012; Hu et al., 2023). This gap matters because early development is a sensitive period during which the immune system can set long-term trajectories, influencing both fledging and adult success (Cam and Aubry, 2011; Lindström, 1999; Metcalfe and Monaghan, 2001). Likewise, studies in natural settings are essential to accurately evaluate the real costs of immune activation, as they reflect the ecological context and environment-driven trade-offs in which such responses operate. In the wild, immune function is shaped by interacting environmental factors – such as food availability and quality, and temperature variation, among others – that affect the balance between immunity and other vital processes (e.g. McNamara and Buchanan, 2005; Zuk and Stoehr, 2002). These interactions are often absent in captivity, where animals are shielded from ecological stressors, potentially underestimating or misrepresenting the true costs of immunity (Costantini and Møller, 2009; Hasselquist and Nilsson, 2012). Furthermore, it should be noted that the costs of mounting an immune response and the individual's capacity to cope with that challenge largely depend on its initial condition (McNamara and Buchanan, 2005). For example, last-hatched nestlings within altricial avian broods, typically smaller and in poorer condition, may suffer from increased oxidative stress (e.g. Montoya et al., 2020; Rubolini et al., 2006; Stier et al., 2015). Indeed, these marginal nestlings often develop weaker immune responses (often measured as T-cell-mediated swelling in response to phytohaemagglutinin) compared with their larger siblings (Banda and Blanco, 2008; Christe et al., 1998; Martínez-Padilla and Viñuela, 2011; Müller, 2003). It is therefore expected that the cost of mounting an immune response will be higher for the smallest nestlings within a brood.

In this study, we investigated the effects of mounting an immune response in wild spotless starling (*Sturnus unicolor* Temminck 1820) nestlings by exposing them to a membrane lipopolysaccharide (LPS) derived from *Escherichia coli*, a commonly used approach (e.g. Ashley and Wingfield, 2012). We specifically analysed the impact of this immune challenge on nestling mass at four different time points after the challenge to detect even small changes in their growth trajectory (Metcalfe, 2024), as well as on oxidative stress markers 24 h after. We tested these effects under a particularly challenging ecological scenario for this species: the second wave of clutches of the breeding season. In our study population, spotless starlings typically lay two clutches per season that develop under contrasting environmental conditions (Salaberria et al., 2014). First-wave clutches (April–May) benefit from higher food availability and milder temperatures, while second-wave clutches (May–June) face food scarcity, thermal stress and reduced parental capacity due to prior breeding efforts, which impairs nestling growth and condition (López-Rull et al., 2010; Muriel et al., 2015; Salaberria et al., 2014). Nestlings from the second wave of clutches also experience higher ectoparasite and endoparasite loads (De la Peña et al., 2024; López-Rull et al., 2010) and higher hatching asynchrony (up to 48 h) in comparison with first-wave broods, leading to greater developmental differences among chicks (Muriel et al., 2015), which contributes to brood reductions. These factors probably increase nestling vulnerability to immune challenges, especially in smaller chicks. If immune response activation leads to the previously mentioned trade-offs against growth and oxidative status, we expect immune-challenged wild nestlings to experience reduced mass and increased oxidative damage. Additionally, we expect these effects to be more severe in the smallest chicks within a brood.

## MATERIALS AND METHODS

All procedures were reviewed by the Animal Experimentation Ethics Committee of the University of Castilla-La Mancha and approved by the competent authority of the Junta de Comunidades de Castilla-La Mancha (reference: 21-2021), thus complying with current European, Spanish and Institutional guidelines for the care and use of animals.

The study was conducted in a nest-box breeding population of spotless starlings located in central Spain (Soto del Real, Madrid). In this population, modal brood size is four nestlings that fledge after 21–22 days (Muriel et al., 2021). We determined the hatching date of each clutch through regular nest inspections.

We selected 121 5-day-old nestlings from 34 nests (brood size range 2–5 nestlings) during the second wave of clutches (late May to late June) in 2021. Body mass of nestlings was recorded (to the nearest 0.1 g) and a blood sample (200 µl) was collected from the jugular vein to quantify pre-treatment levels of haptoglobin and oxidative stress markers (see below). The next day (day 6 of age), body mass was recorded again, and nestlings were ranked according to their mass within the brood and assigned to either immune challenge (LPS; *N*=61 nestlings) or control (phosphate-buffered saline, PBS; *N*=60 nestlings) treatments. The treatment was alternated within the brood, and the order of treatments was alternated between broods to avoid bias regarding hatching order and brood sizes. Nestlings in the LPS treatment received an intraperitoneal injection of 1.25 ml kg^−1^ LPS (ref: L2880, Sigma-Aldrich) suspended in PBS at a concentration of 0.8 mg ml^−1^. This is equivalent to a dose of 1 mg kg^−1^ of body mass. Control nestlings received the same volume of PBS alone. This dosage was selected based on a previous experiment conducted in nestlings from the same population and age (Parejo-Pulido et al., 2024), as well as on dose-dependent results reported in northern bobwhite quails (*Colinus virginianus*) (Armour et al., 2020), where this dose generated the most pronounced physiological responses, but not deleterious effects on survival. Six hours later, we returned to the nests and recorded nestling mass to investigate very short-term effects of the treatment on body mass.

The next morning (day 7 of age), 24 h after the LPS injection, we weighed the chicks and collected a second blood sample to assess the effect of the treatment on haptoglobin and oxidative stress markers. A drop of blood from the collected sample was stored in 96% ethanol for molecular sexing. Blood samples from day 5 and 7 were stored at 4°C until centrifugation (5 min at 4°C and 9000 ***g***) within 5 h after collection. Plasma was separated from erythrocytes and frozen at −80°C until analysis. Nestlings were weighed again on days 8 and 14 to explore the mid-term effect of treatment on body mass.

### Haptoglobin assay

To validate the effectiveness of our treatment to activate the immune system, we analysed haptoglobin levels in a random subset of 29 nestlings from nine nests (17 LPS and 12 control nestlings). Haptoglobin is an acute phase protein that increases rapidly in response to infection, inflammation or trauma, such as that produced by LPS (Millet et al., 2007). We used a commercial colorimetric kit (TP801, Tridelta Development Ltd) following the manufacturer's instructions with minor modifications described in detail in Parejo-Pulido et al. (2025). To each well of 96-well flat-bottom plates, we added 6 μl of plasma sample and 80 μl of reagent 1, vortexed and recorded absorbance at 450 and 630 nm using a microplate reader (Thermo Scientific Multiskan GO). We then added 112 μl of reagent 2, incubated the plates at 22°C for 5 min and took a final absorbance measurement at 630 nm. We subtracted the initial 630 nm absorbance (before adding reagent 2) from the post-incubation measurement at 630 nm absorbance to correct for plasma turbidity. The 450 nm pre-scan enabled us to statistically analyse and correct for differences in plasma sample redness, indicating sample haemolysis, which can affect assay results (Matson et al., 2012). We calculated haptoglobin concentration (mg ml^−1^) from the difference in absorbance at 630 nm between the pre- and post-incubation measurements, using a standard curve quantified on each plate. We assessed the repeatability using a set of 15 random samples analysed in duplicate [intraclass correlation coefficient (ICC)=0.703, *F*_14,14_=5.42, *P*=0.002].

### Oxidative damage in lipids

We measured plasma levels of malondialdehyde acid (MDA), a widely used marker of oxidative damage to lipids and a proxy for whole-body oxidative damage (e.g. Margaritelis et al., 2015). We quantified MDA using high-performance liquid chromatography (HPLC) following protocols recently published (Parejo-Pulido et al., 2025). Briefly, we added 50 μl of butylated hydroxytoluene (0.05% w/v in 95% ethanol), 400 μl of 0.44 mol l^−1^ phosphoric acid, 100 μl of 42 mmol l^−1^ thiobarbituric acid (TBA) and 30 μl of distilled water to 20 μl of each plasma sample. After incubating at 100°C for 1 h, we extracted the TBA-MDA adducts formed with 250 μl of *n*-butanol, and quantified MDA levels in an Agilent 1200 series HPLC system with a fluorescence detector set at 515 nm (excitation) and 553 nm (emission). We used a C18 column (5 mm ODS-2 4.0×250 mm) at 37°C and an isocratic flow of methanol:KH_2_PO_4_ (50 mmol l^−1^; 40:60 v/v) at 1 ml min^−1^. We used an 8-point serial dilution of 10 μmol l^−1^ 1,1,3,3-tetraethoxypropane as the calibration curve. Data are reported in µmol of MDA per litre of plasma. The repeatability of this technique was assessed using a subset of 18 samples extracted and analysed in duplicate (ICC=0.80, *F*_17,17_=8.61, *P*<0.001).

### Reactive oxygen metabolites

We measured the concentration of reactive oxygen metabolites (ROMs) in plasma samples using the d-ROMs test (Diacron, Grosseto, Italy). The d-ROMs test quantifies hydroperoxides, which are oxidatively damaged biomolecules such as polyunsaturated fatty acids, cholesterol, proteins and nucleic acids and are precursors of end products of lipid peroxidation, such as MDA (Costantini, 2016). We followed the manufacturer's instructions for the kinetic version of the assay with minor modifications. In short, we pipetted 20 µl of each plasma sample into each well of a 96-well flat-bottom microplate. In duplicate with each batch of samples, we also pipetted 10 µl and 20 µl of the kit calibrator and blank (ultrapure water), respectively. We then added 180 µl of commercial reagent 2 to all wells, briefly vortexed the plate, and immediately measured absorbance at 546 nm using the same microplate reader mentioned above. Next, we added 20 µl of the chromogen (diluted 1:10 in ultrapure water), briefly vortexed the plate again, incubated it in darkness at 37°C for 75 min, and took a post-incubation measurement at 546 nm. To calculate the final values, we subtracted the initial measurement (before adding the chromogen) from the post-incubation measurement to correct for plasma colour and turbidity. Absorbance was then converted to mg H_2_O_2_ dl^−1^ according to the manufacturer's instructions. A subset of nine samples was quantified in duplicate to calculate the repeatability of the technique (ICC=0.76, *F*_8,8_=9.03, *P*=0.005).

### Antioxidant capacity of plasma

The antioxidant capacity of plasma samples was evaluated using the OXY-adsorbent Assay (Diacron) following the protocol described in detail in Parejo-Pulido et al. (2025). The test measures the plasma non-enzymatic antioxidant capacity (OXY) of both exogenous (e.g. ascorbate, tocopherols, carotenoids, bioflavonoids) and endogenous (e.g. albumin, uric acid, reduced glutathione) compounds. We pipetted 10 µl of blank (ultrapure water), calibrator or plasma sample (the last two diluted 1:100 in ultrapure water) into each well of a 96-well flat-bottom microplate. We then added 150 μl of HClO solution to each well, vortexed and measured the absorbance at 546 nm with the same microplate reader mentioned above. We incubated the plate for 10 min at 37°C, added 50 μl of chromogen (diluted 1:10 in ultrapure water) and measured the absorbance again at 546 nm. The intensity of the pink derivative depends on the residual HClO not neutralized by antioxidants in the sample, therefore inversely reflecting the antioxidant power of the sample. This initial absorbance was subtracted from the final measurements to control for sample turbidity for the final calculation of antioxidant capacity, which is expressed as µmol of HClO neutralized. We confirmed the repeatability of this technique using a subset of 18 samples (ICC=0.959, *F*_17,15_=54.5, *P*<0.001).

### Triglycerides

MDA and ROM values are highly sensitive to plasma triglycerides, making it advisable to statistically control for them (Pérez-Rodríguez et al., 2015). We quantified this metabolite using a commercial kit (ref. 11,529, Biosystems, Barcelona, Spain) based on the glycerol phosphate oxidase/peroxidase method. We followed the manufacturer's instructions, with minor modifications described in Parejo-Pulido et al. (2025) to adapt the technique to low sample volumes and 96-well microplates. A subset of 16 samples was assayed in duplicate to calculate repeatability (ICC=0.671, *F*_15,16_=5, *P*=0.001).

### Statistical analyses

We performed statistical analyses using R 4.4.1 (http://www.R-project.org/). To enhance model stability, the likelihood of model convergence and the accuracy of variable estimates, all independent variables were *Z*-transformed (mean-centred with a s.d. of 1) (Harrison et al., 2018). Model assumptions were checked using the packages *DHARMa* (https://CRAN.R-project.org/package=DHARMa) and *performance* (Lüdecke et al., 2021).

The effect of the immune challenge on growth was assessed using two approaches depending on the time elapsed since the LPS/PBS injection. Firstly, we analysed short-term effects on nestling growth by analysing body mass over the first 48 h after the treatment, as a recent study in this species has shown that LPS can affect growth specifically during this period under laboratory conditions (Parejo-Pulido et al., 2024). We used a (repeated measures) linear mixed model with body mass as the response variable and the interaction between post-treatment time [four levels: 0 (i.e. just before injection), 6 h, 24 h and 48 h after treatment] and treatment (LPS or PBS) as the main predictors. We also included nestling sex, brood size and the date of the experiment as covariates, and nestling ID nested within nest of origin as random effects. We conducted multiple pairwise comparisons between time post-treatment points using Tukey's *post hoc* test in the R package *emmeans* (https://CRAN.R-project.org/package=emmeans).

Secondly, we separately analysed medium-term effects on growth by analysing differences between treatment groups in body mass at day 14 using a linear mixed model. Body mass at this age is a key trait in our study system as it represents fledgling size and predicts reproductive success at adulthood (Redondo et al., 2022). In addition, we assessed whether the probability of surviving until day 14 was influenced by the treatment using a generalized mixed model with a binomial error distribution (1=alive, 0=dead). In both models, the treatment was included as the main predictor and initial body mass, nestling sex, brood size and date as covariates. Nest was included as a random effect. As differences in mass among chicks may reflect variation in their ability to mount an immune response (see Introduction), we tested the interaction between initial nestling mass and treatment. If this interaction was non-significant, it was removed from the final model. To further explore whether treatment effects on body mass and survival probability at day 14 varied according to the initial body mass of nestlings, we divided nestlings into quartiles (Q1–Q4) based on their initial body mass. We then focused on the two extreme quartiles –Q1 (smallest nestlings) and Q4 (largest nestlings) – and analysed them separately, as treatment effects were expected to differ mainly at the extremes of the mass distribution. For each quartile, we fitted a linear (for body mass at day 14) or a generalized (for survival probability until day 14) mixed effects model with treatment, initial mass (and their interaction), sex, hatching date and brood size as fixed effects, and nest as a random effect.

We then assessed the effect of the immune challenge on nestling physiology (haptoglobin, MDA, ROMs and OXY levels) within 24 h post-treatment using linear mixed models (haptoglobin, MDA, OXY) or a generalized mixed model (ROMs). For the linear mixed models of haptoglobin, MDA and OXY levels, experimental treatment was the main predictor, while nestling sex, brood size, the date of the experiment, body mass just before the injection (initial mass on day 6) and initial pre-treatment values of each physiological marker – to control for pre-treatment levels – were included as covariates. Nest was included as a random effect. In the haptoglobin model, we also included the 450 nm pre-scan absorbance value (see Materials and Methods) at 24 h post-treatment as a covariate; and calculated initial pre-treatment haptoglobin values as residuals after controlling for their 450 nm pre-scan absorbance. ROMs levels were analysed using a generalized mixed model with a binomial error distribution because a significant portion of the data (160 out of 225 measures) showed no detectable levels. Thus, for this particular variable, we analysed the probability of showing detectable levels of ROMs. As in the models for body mass at day 14 and survival probability, we also tested the interaction between initial mass and treatment and removed it if it was not significant. Alternative models for MDA and ROMs including triglyceride levels as an extra explanatory variable were also run (Pérez-Rodríguez et al., 2015). Furthermore, to distinguish the effects of the immune challenge itself from those of potential compensatory growth on oxidative marker levels (as suggested by Metcalfe and Monaghan, 2001; Stier et al., 2014, 2015), we conducted alternative models that included mass gain during the period of most rapid growth in LPS nestlings (see Results) as an additional explanatory variable.

In all models described above, we tested the three-way interaction among treatment, time post-treatment or body initial mass, and sex, as well as the two-way interactions between treatment and sex, and between time post-treatment or initial body mass and sex. As none of these interaction terms were significant, we excluded them from the final models. It is worth noting that, although sexual differences in body mass are well documented in nestlings of this species (e.g. Muriel et al., 2021), and the inclusion of both variables (sex and initial mass) could raise concerns about potential collinearity, we verified model assumptions and collinearity. Specifically, we calculated the variance inflation factors (VIF) for all predictors. In all models, the VIF for sex was consistently below 2, confirming that the statistical effect of sex was independent of initial body mass and other predictors.

To address multiple testing when analysing how experimental treatment affected multiple physiological variables, we applied a *P*-value correction using the Benjamini–Hochberg method to control for false discovery rate in growth and oxidative stress analyses (Benjamini and Hochberg, 1995; Storey and Tibshirani, 2003).

## RESULTS

### Body mass dynamic and survival

**Fig. 1.**
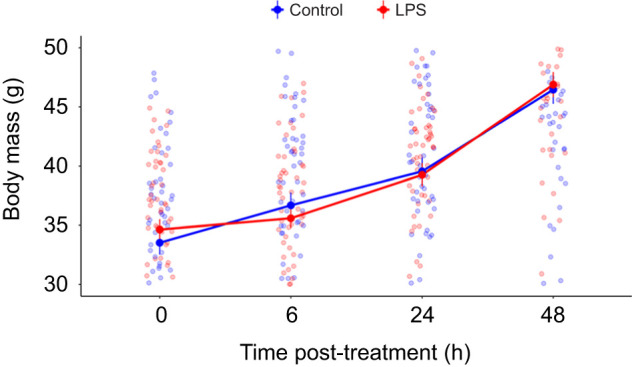
**Body mass of lipopolysaccharide (LPS)-treated and control nestlings just before eliciting the immune response (hour 0) and 6, 24 and 48 h after the immune challenge.** Data are means±s.e.m. Points represent raw values for each individual.

We found no differences in body mass among experimental groups before the immune challenge (day 5 of age: *t*_1,88_=0.22, *P*=0.490). However, LPS treatment significantly affected nestling body mass changes during the first 48 h post-treatment (Table 1). *Post hoc* comparisons showed no significant differences in overall body mass between LPS and control nestlings at any individual sampling point (all *P*>0.964; Table S1). However, the pattern of body mass gain differed between the groups (Fig. 1; Table S1). Whereas control nestlings showed a constant significant increase in body mass over the entire 48 h period (i.e. from 0 to 6 h, from 6 h to 24 h and from 24 h to 48 h; all *P*<0.001), LPS nestlings showed two clearly distinct phases during this 48 h period after treatment (Fig. 1). During the first 6 h, LPS nestlings did not significantly increase their body mass (*P*=0.337; Table S1), whereas they showed significant body mass gain between 6 and 48 h post-treatment (i.e. from 6 h to 24 h and from 24 h to 48 h; all *P*<0.001). Indeed, this increase in body mass of LPS nestlings between 6 and 24 h post-treatment was more pronounced compared with that of the controls (treatment effect on mass at 24 h post-treatment after controlling for body mass at 6 h post-treatment: estimate±s.e.=0.85±0.39, *t*_1,85_=2.17, *P*=0.033; Table S2).

**Table 1.** Effect of experimental treatment [lipopolysaccharide (LPS) or control] on nestling body mass at 48 h post-treatment and at day 14 of age, and on the probability of survival until day 14

	*F*	χ^2^	d.f.	*P*
**48 h growth**				
Brood size	1.72		1, 37	0.198
Date	5.61		1, 34	0.024
Sex	5.28		1, 107	0.023
Treatment	0.42		1, 105	0.516
Hours	321		3, 348	<0.001
Treatment×Time	4.67		3, 348	**0.003**
**Body mass day 14**				
Brood size	4.14		1, 28	0.052
Date	0.08		1, 28	0.785
Sex	4.48		1, 79	0.037
Initial mass	16.4		1, 81	<0.001
Treatment	2.31		1, 67	0.133
Treatment×Initial mass	6.20		1, 72	**0.015**
**Survival probability until day 14**				
Brood size		1.72	1	0.190
Date		0.47	1	0.493
Sex		4.49	1	0.034
Initial mass		8.91	1	0.003
Treatment		3.99	1	0.046
Treatment×Initial mass		4.40	1	**0.036**

*F*-values, d.f. and *P*-values for all fixed effects are shown. For the generalized linear mixed models analysing the probability of survival, we provide χ^2^ values instead of *F*-values. Significant *P*-values for the treatment effect are in bold.

**Fig. 2.**
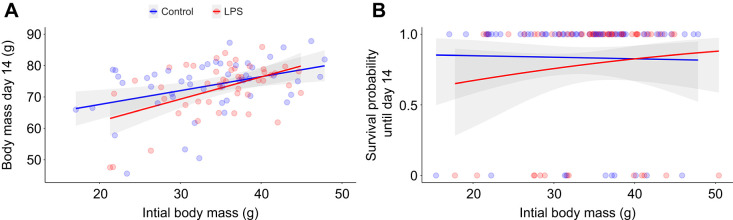
**Relationship between initial (day 6) body mass and day 14 body mass and survival probability for LPS-treated and control nestlings.** (A) Body mass at day 14 and (B) survival probability until day 14. Points represent raw values for each individual. Grey bands represent 95% confidence interval for the regression lines of each group.

At day 14 of age (8 days post-treatment), the effects of the treatment on body mass depended on the initial mass (Table 1, Fig. 2A). The relationship between body mass at day 14 of age and initial mass was stronger in LPS-treated nestlings than in control nestlings (LPS nestlings: estimate±s.e.=6.11±1.18, *t*_1,39_=5.18, *P*<0.001; control nestlings: estimate±s.e.=3.13±0.93, *t*_1,31_=3.37, *P*=0.002). This occurred because LPS nestlings that started the experiment with a low initial mass attained less body mass at day 14 compared with control nestlings with similar initial mass. This interpretation was supported by separate analyses of nestlings in the lowest (Q1) and highest (Q4) initial mass quartiles. Treatment had no significant effect on the heaviest nestlings (estimate±s.e.=−0.95±2.19, *t*_1,17_=−0.43, *P*=0.672; Table S3). In contrast, among the smallest nestlings, we found a significant treatment×initial mass interaction (Table S3): among LPS-treated nestlings, the slope relating initial mass to day 14 mass was markedly steeper than in control nestlings (estimate±s.e.=25.0±9.18, *t*_1,17_=2.72, *P*=0.014; Fig. S1). In practice, the very smallest LPS-treated nestlings gained little additional mass until day 14, whereas the heavier individuals within Q1 grew as much as control nestlings. These results confirmed that the effect of LPS on mass at day 14 is confined to the smallest nestlings.

The same significant interaction (treatment×initial mass) was found when analysing the survival probability until day 14 (Table 1, Fig. 2B). Survival probability was significantly and positively related to initial mass in LPS-treated nestlings (estimate±s.e.=11.4±3.32, *z*=3.43, *P*<0.001), but not in control nestlings (estimate±s.e.=1.46±2.48, *z*=0.59, *P*=0.556). By day 14, 21.3% (13/61) LPS and 16.6% (10/60) control nestlings had died, which does not represent an overall significant difference among treatments (Fisher exact test, *P=*0.64). However, LPS chicks that started the experiment with low body mass were more likely to die than controls of similar initial mass. LPS-treated nestlings in Q1 showed a significantly lower survival probability than control nestlings (estimate±s.e.=−56.5±13.1, *z*=−4.31, *P*<0.001), whereas treatment had no significant effect on survival in Q4 (estimate±s.e.=−0.61±5.64, *z*=−0.11, *P*=0.913) (Table S3). Thus, facing an immune challenge exacerbated the impact of initial size on body mass a few days before fledging, and decreased the survival prospects of small individuals during the nestling period.

We also found that female nestlings always showed lower body mass (body mass during 48 h post-treatment: estimate±s.e.=−3.06±1.33, *t*_1,107_=−2.30, *P*=0.023; body mass at day 14: estimate±s.e.=−2.88±1.36, *t*_1,79_=−2.12, *P*=0.038) and chances of survival until day 14 (estimate±s.e.=−8.07±3.68, *z*=−2.19, *P*=0.029) than males irrespective of age and treatment (Table 1).

### Haptoglobins

**Fig. 3.**
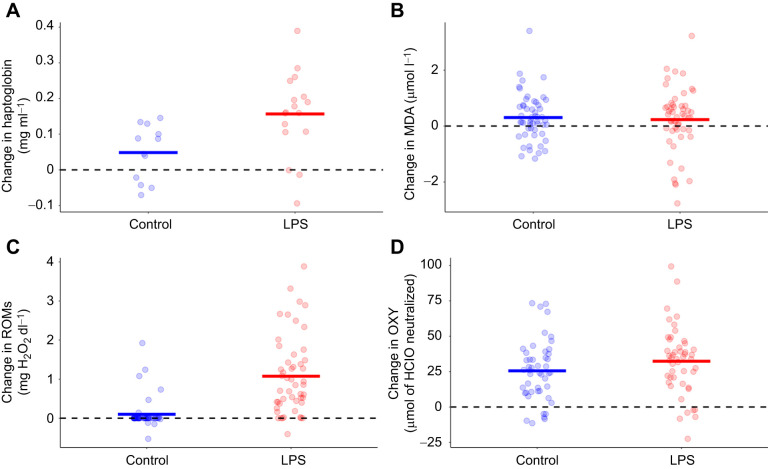
**Effect of experimental treatment (LPS or control) on nestling physiology.** The change in (A) haptoglobin, (B) malondialdehyde acid (MDA), (C) reactive oxygen metabolites (ROMs) and (D) antioxidant capacity (OXY) levels. Each point is the raw difference between post- and pre-treatment values for each individual. Bold lines are group mean changes. Dashed lines indicate no change in each variable between pre- and post-treatment measurements. In the case of ROMs, for the calculation of the change, we assigned a value of zero to samples with no detectable levels.

Our experimental manipulation successfully activated the immune response, as LPS nestlings exhibited higher levels of haptoglobins than controls 24 h after the challenge (estimate±s.e.=0.10±0.03; *t*_1,17_=3.19, *P*=0.005; Table 2, Fig. 3A).

**Table 2.** **Effect of experimental treatment (LPS or control) on 7** **day nestling plasma haptoglobin levels, plasma malondialdehyde acid (MDA), the probability of showing detectable levels of reactive oxygen metabolites (ROMs) in plasma and plasma antioxidant capacity (OXY) post-treatment levels considering initial pre-treatment values**

	*F*	χ^2^	d.f.	*P*	Corrected *P*
**Haptoglobin**					
Brood size	0.04		1, 4	0.851	** **
Date	0.06		1, 5	0.821	
Sex	0.03		1, 21	0.859	
Initial mass	0.44		1, 21	0.514	
Pre-scan absorbance at 450 nm	2.20		1, 21	0.153	
Initial haptoglobin (residuals)	2.88		1, 21	0.105	
Treatment	10.2		1, 17	**0.005**	**0.005**
**MDA**					
Brood size	0.40		1, 32	0.529	** **
Date	0.11		1, 31	0.742	
Sex	0.20		1, 82	0.656	
Initial mass	4.22		1, 93	0.043	
Initial MDA	33.6		1, 75	<0.001	
Treatment	0.04		1, 72	0.839	0.839
**ROMs (prob)**					
Brood size		0.09	1	0.759	
Date		0.01	1	0.919	
Sex		0.74	1	0.391	
Initial mass		1.75	1	0.186	
Initial ROMs (prob)		0.42	1	0.519	
** **Treatment		10.8	1	**<0.001**	**0.001**
**OXY**					
Brood size	0.21		1, 28	0.650	
Date	0.05		1, 30	0.826	
Sex	2.57		1, 68	0.113	
Initial mass	8.68		1, 76	0.004	
Initial OXY	34.1		1, 82	<0.001	
Treatment	14.6		1, 63	**<0.001**	**0.001**

*F*-values, d.f. and *P*-values for all fixed effects are shown. We also provide corrected *P*-values (Benjamini–Hochberg method) for the treatment. For generalized linear mixed models analysing the probability of survival, we provide χ^2^ values instead of *F*-values. Significant *P*-values for the treatment effect are in bold.

### Oxidative stress

There were no initial differences in any of the oxidative markers analysed between groups (corrected *P*>0.293 in all cases; Table S4). Twenty-four hours after the immune challenge, LPS nestlings exhibited a higher probability of showing detectable oxidative damage (ROMs; estimate±s.e.=4.68±1.42, *z*=3.29, *P*<0.001, corrected *P*=0.001) and higher plasma antioxidant capacity (OXY; estimate±s.e.=8.11±2.12, *t*_1,63_=3.82, *P*<0.001, corrected *P*=0.001) compared with controls (Table 2, Fig. 3C,D). This increase in ROM levels resulted solely from the immune challenge (corrected *P*=0.003; Table S5) and was independent of the rapid mass gain between 6 and 24 h post-challenge observed in LPS nestlings (corrected *P*=0.782; Table S5). In contrast, the increase in OXY levels was driven by both the immune challenge (estimate±s.e.=6.56±2.09, *t*_1,64_=3.14, *P*<0.003, corrected *P*=0.004; Table S5) and the accelerated mass gain during that period (estimate±s.e.=5.29±1.53, *t*_1,87_=3.46, *P*=0.001, corrected *P*=0.003; Table S5). However, we found no significant effects of the immune challenge or mass gain between 6 and 24 h post-challenge on levels of oxidative damage to plasma lipids (MDA; Table 2, Fig. 3B; Table S5). The results did not change when triglyceride levels were included as an extra covariate on MDA or ROMs models (Table S6).

## DISCUSSION

In this study, we induced an experimental immune challenge using LPS to analyse the effects of immune response on growth dynamics and oxidative stress in developing wild spotless starling nestlings. Our results suggest that the activation of the immune response temporarily compromises nestling growth dynamics, as evidenced by reduced mass gain exclusively within the first 6 h following the immune challenge. This impairment was particularly disruptive for the smallest nestlings in the broods, reducing both their probability of surviving until fledging and their body mass at the end of the nestling stage. The induced immune response also caused an increase in oxidative damage and antioxidant capacity, supporting a link between immune activation and oxidative stress during development in wild vertebrates.

In altricial birds, the body mass achieved before fledging strongly predicts later survival and reproductive success (Magrath, 1991). In spotless starlings in particular, body condition at the end of the growth period predicts the probability of reproduction during the first years of adulthood (Redondo et al., 2022). Thus, any factor that disrupts or constrains growth may have direct negative consequences on the fitness of individuals. We found that simulating an infection through LPS administration during the early days of life of wild spotless starling nestlings affected both growth dynamics as well as survival probability until fledging, depending on the initial body mass. In a first step, LPS treatment negatively affected mass dynamic, with LPS nestlings showing no increase in body mass in the first 6 h after treatment compared with control nestlings. Two different scenarios may explain this lack of body mass increase. First, mounting an immune response may involve physiological trade-offs that temporarily divert resources from growth, as observed in many previous studies (e.g. Armour et al., 2020; Bonneaud et al., 2003; Burness et al., 2010; Merino et al., 2000; Soler et al., 2003; Schneeberger et al., 2013). Immune activation is highly resource demanding, and as a result, nestlings must reallocate their limited resources, prioritizing recovery and survival over growth. Second, our results are also consistent with the known effects of the acute-phase response to infection, which reduces food intake as a result of suppressed appetite in the hours following immune response activation (Hart, 1988; Owen-Ashley and Wingfield, 2007; Parejo-Pulido et al., 2024). Although reduced feeding may seem counterproductive, it is considered an adaptive strategy that optimizes immune function until recovery (Hart, 1988; Kyriazakis et al., 1998). As a consequence, they may also reduce their begging behaviour (Parejo-Pulido et al., 2024), which probably affects how often their parents feed them.

However, after this 6 h of no growth, LPS nestlings exhibited accelerated growth, gaining mass faster than control nestlings between 6 and 24 h post-treatment. Indeed, by 24 h, the two groups had reached similar mass and continued to gain mass at comparable rates up to 48 h. This compensatory growth pattern, also known as ‘catch-up’ growth, has been observed in several species as a strategy to recover from mass loss during periods of nutritional deficit (Hector and Nakagawa, 2012; Metcalfe and Monaghan, 2001, 2003). This shift in physiological priorities could result from a reduction in the resources allocated to immune function, as the initial production phase of mounting an immune response is thought to be resource intensive, but maintaining it requires fewer resources (Derting and Compton, 2003). Additionally, if the acute effects of LPS during the acute-phase response, such as reduced appetite, have subsided by this time, nestlings might have intensified their begging behaviour to obtain more food from their parents, as observed in female barn swallow (*Hirundo rustica*) nestlings 24 h after LPS administration (Romano et al., 2011). Either way, the growth dynamic of LPS nestlings perfectly illustrates an ‘adjusted trajectory’ strategy (see figure 1B in Metcalfe, 2024), where the ultimate target remains unaltered (i.e. body mass by day 8), but the developmental trajectory to reach it is affected. The rapid development of this strategy within such a short time period may explain some discrepancies in the literature, where less detailed body mass monitoring failed to detect differences between control and immune-challenged nestlings [for example, in the European starling (*Sturnus vulgaris*) Serra et al. (2012), or in broiler chickens Zheng et al. (2020)]. Both ‘catch-up’ growth and the ‘adjusted growth trajectory’, while necessary, may incur costs later on (Metcalfe and Monaghan, 2001; Metcalfe, 2024). Indeed, ‘catch-up’ growth has been shown to lead to a sudden overproduction of reactive oxygen species, which could overwhelm the chicks’ antioxidant defence and generate oxidative damage (Stier et al., 2014, 2015).

In this sense, we found that both the immune response triggered by LPS and the rapid mass gain between 6 and 24 h post-challenge increased antioxidant capacity (OXY) 24 h after the immune challenge. We also observed an increase in oxidative damage (ROMs), but this was solely generated by the immune challenge, not by the rapid growth. In contrast, oxidative damage to lipids (MDA) remained unaffected by either factor. Although these results contrast with those of Serra et al. (2012), who reported no effects of LPS on ROM levels or antioxidant capacity in nestlings of the closely related European starling, our findings support the oxidative cost of the immune response (meta-analysis in Costantini, 2022). Several studies have shown that immune cell activity during an immune challenge increases ROMs (e.g. Armour et al., 2020; Costantini and Dell'Omo, 2006; van de Crommenacker et al., 2010). In contrast, MDA is used less frequently (Costantini, 2022; but see Hu et al., 2023, in poultry chicks and Alejandro et al., 2025, for a recent study on quails). Differences between these markers probably stem from the distinct oxidative products they measure and their varying sensitivity to immune responses. While the quantification of MDA levels specifically measures lipid peroxidation (Monaghan et al., 2009), the d-ROM test measures oxidized biomolecules (hydroperoxides) at earlier stages of the cascade of oxidative reactions in a more unspecific way, including polyunsaturated fatty acids, cholesterol, proteins and nucleic acids (Costantini, 2016). Thus, although both ROMs and MDA are biomarkers of oxidative damage, they may represent distinct aspects of this process that may be affected differently by the functioning of the immune response. In addition, LPS nestlings and those that gained mass faster between 6 and 24 h post-challenge showed an increase in their plasma antioxidant capacity, which might be explained as an organism response to counteract the damage caused by the immune response and the rapid growth.

Finally, we found that 6 days after the experimental manipulation (day 14 of age), the effect of initial mass on body mass differed between LPS and control groups. LPS nestlings that started the immune challenge with lower mass, gained less mass than control nestlings of similar initial mass by day 14. Additionally, these smaller LPS nestlings had a higher likelihood of dying. Spotless starlings exhibit a moderate degree of hatching asynchrony, with these smallest nestlings typically hatching later and from last-laid eggs (Gil et al., 2008; Muriel et al., 2015). This leads to significant differences in body mass and size among siblings (Muriel et al., 2015) that are also associated with other physiological aspects, such as DNA integrity in blood cells (Montoya et al., 2020). These differences would be magnified in second-wave clutches, when this experiment was performed, as environmental conditions are harsher (Gil et al., 2019; Salaberria et al., 2014) and parents are more likely to prioritize the most viable, bigger offspring (Caro et al., 2016). Indeed, we found that females – the smaller and less competitive sex in this sexually size dimorphic species (Muriel et al., 2021) – showed lower overall survival until day 14. This is consistent with previous findings in our study population showing that female nestlings experienced higher levels of stress (inferred from circulating corticosterone) when sibling competition was experimentally increased (Gil et al., 2019).

In conclusion, our findings highlight the detrimental effects of mounting an immune response on growth dynamics, survival and oxidative damage in developing wild birds, providing evidence that nestlings can adjust their growth trajectories (i.e. an adjusted trajectory of no growth followed by a catch-up growth) within a remarkably short time frame. This underscores the importance of closely monitoring these dynamic processes, as they can change within hours, and may help explain the lack of effects observed in some studies. Finally, our study emphasizes the significance of individual body condition in determining how organisms deal with challenges.

## Supplementary Material

10.1242/jexbio.250556_sup1 Supplementary information
