# Supplementary material for: An immune challenge affects growth dynamics, oxidative stress and survival in wild spotless starling nestlings
Source: J Exp Biol. 2025 Aug 4;228(15):jeb250556. doi: 10.1242/jeb.250556 (PMC12377811; doi:10.1242/jeb.250556)
Supplement: Supplementary information [file jexbio-228-250556-s1.pdf]

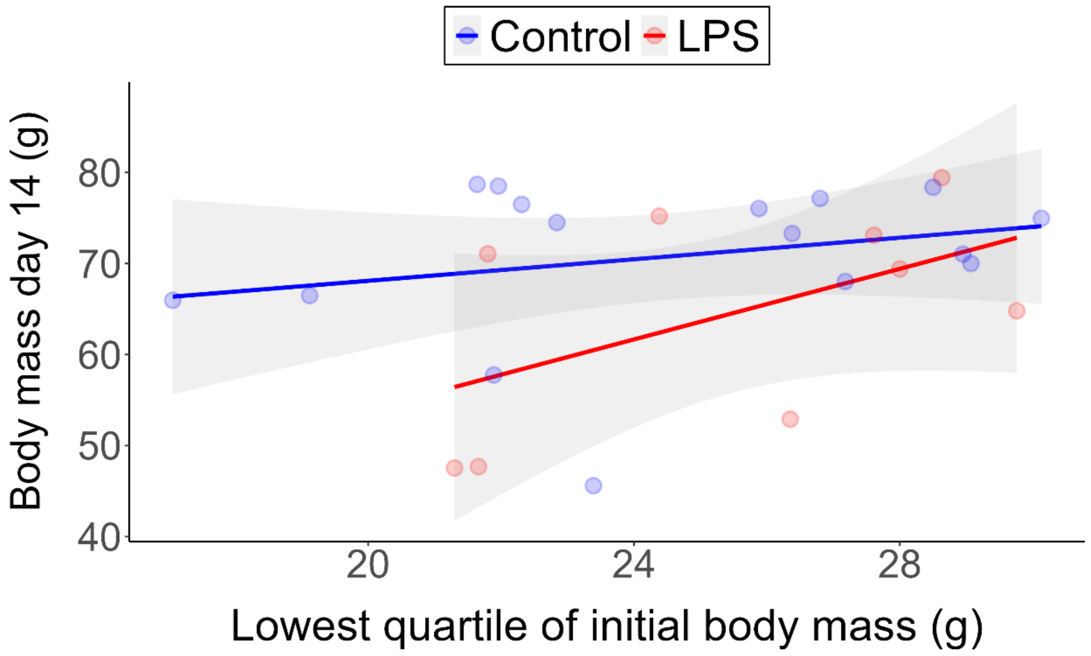

Fig. S1. Relationship between the lowest quartile of initial body mass and body mass at day 14 for LPS (red) and Control (blue) nestlings. Points represent raw values for each individual. Grey bands represent 95% CI for the regression lines of each group.

Journal of Experimental Biology • Supplementary information

Table S1. Post-hoc comparisons (Tukey test) of body mass differences between LPS and Control nestlings and mass gain over 48h post-treatment (Overall effect $F_{3,\ 348} = 4.67$, P = 0.003). We show estimates for each contrast (± SE), t ratio values and *P*-values.

	Estimate	SE	*t*	*P*
PBS initial – LPS initial	-0.85	1.31	-0.65	0.998
PBS 6h – LPS 6h	1.38	1.31	1.06	0.964
PBS 24h – LPS 24h	0.62	1.31	0.48	0.999
PBS 48h – LPS 48h	0.57	1.31	0.43	0.999
PBS initial – PBS 6h	-3.18	0.43	-7.33	<0.001
PBS 6h – PBS 24h	-2.91	0.43	-6.71	<0.001
PBS 24h – PBS 48h	-7.07	0.44	-16.0	<0.001
LPS initial – LPS 6h	-0.95	0.43	-2.23	0.337
LPS 6h – LPS 24h	-3.67	0.43	-8.59	<0.001
LPS 24h – LPS 48h	-7.13	0.43	-16.4	<0.001

Table S2. Effect of experimental treatment (LPS or Control) on nestling body mass at 24h post-treatment controlling for mass at 6h post-treatment. We show F and df, and *P*-values for all fixed effects. Significant *P*-value for the treatment effect are in bold.

	F	df	*P*
Brood size	0.67	1, 33	0.417
Date	0.05	1, 32	0.820
Sex	0.14	1, 94	0.705
Mass 6h	996	1, 105	<0.001
**Treatment**	4.71	1, 85	**0.033**

Table S3. Effect of experimental treatment (LPS or Control) on nestling body mass at day 14 for smallest nestlings in the experiment (Q1, lowest quartile of initial mass) and heaviest nestlings (Q4, highest quartile of initial mass). We show F and df, and *P*-values for all fixed effects. For the generalized linear mixed models analysing the probability of survival, we provide χ2 instead of F. Significant *P*-value for the treatment effect are in bold.

	F	χ2	df	*P*
**Body mass day 14**				
**Q1: Smallest nestlings**				
Brood size	0.96		1, 15	0.344
Date	0.22		1, 15	0.643
Sex	2.40		1, 13	0.705
Initial mass	0.01		1, 16	0.923
Treatment	4.72		1, 16	0.045
**Treatment × Initial mass**	7.41		1, 17	**0.014**
**Q2: Heaviest nestlings**				
Brood size	5.54		1, 8	0.048
Date	0.01		1, 11	0.918
Sex	2.67		1, 19	0.119
Initial mass	0.04		1, 14	0.841
Treatment	0.19		1, 14	0.672
**Survival probability until day 14**				
**Q1: Smallest nestlings**				
Brood size		15.0	1	<0.001
Date		<0.01	1	0.953
Sex		7.92	1	0.005
Initial mass		<0.01	1	0.981
**Treatment**		18.5	1	**<0.001**
**Q2: Heaviest nestlings**				
Brood size		0.01	1	0.933
Date		0.02	1	0.888
Sex		<0.01	1	0.946
Initial mass		0.01	1	0.927
Treatment		0.01	1	0.913

Journal of Experimental Biology • Supplementary information

Table S4. Pre-treatment (i.e., day 6 of age) differences in MDA levels, the probability of showing detectable levels of ROMs in plasma, and plasma OXY levels of LPS and Control nestlings. We show F and df, and *P*-values for all fixed effects. We also provide corrected *P*-values (Benjamini-Hochberg method) for the effect of the treatment. For the generalized linear mixed models analysing ROMs, we provide $\chi 2$ instead of F.

	F	χ2	df	*P*	Corrected *P*
**MDA**					
Brood size	4.06		1, 29	0.053	
Date	2.57		1, 29	0.120	
Sex	<0.01		1, 76	0.952	
Initial mass	4.56		1, 80	0.036	
Treatment	2.54		1, 71	0.115	0.294
**ROMs (prob)**					
Brood size		0.05	1	0.825	
Date		0.17	1	0.678	
Sex		0.31	1	0.578	
Initial mass		0.55	1	0.460	
Treatment		1.67	1	0.196	0.294
**OXY**					
Brood size	0.35		1, 29	0.557	
Date	5.08		1, 30	0.032	
Sex	0.01		1, 76	0.912	
Initial mass	15.0		1, 86	<0.001	
Treatment	0.01		1, 65	0.936	0.936

Journal of Experimental Biology • Supplementary information

Table S5. Effect of experimental treatment (LPS or Control) on MDA levels, the probability of showing detectable levels of ROMs in plasma, and plasma OXY levels of LPS and Control nestlings, considering the mass gain between 6 and 24h post-challenge. We show F and df, and *P*-values for all fixed effects. We also provide corrected *P*-values (Benjamini-Hochberg method) for the effect of the treatment and the mass gain between 6 and 24h post-challenge. For the generalized linear mixed models analysing ROMs, we provide χ2 instead of F. Significant *P*-value for the treatment and the mass gain between 6 and 24h post-challenge effects are in bold.

	F	χ2	df	*P*	Corrected *P*
**MDA**					
Brood size	0.42		1, 32	0.519	
Date	0.11		1, 31	0.742	
Sex	0.19		1, 82	0.665	
Initial mass	3.74		1, 93	0.056	
Mass gain 6-24h post-challenge	0.08		1, 93	0.782	0.782
Treatment	0.02		1, 72	0.881	0.881
**ROMs (prob)**					
Brood size		0.10	1	0.748	
Date		0.01	1	0.912	
Sex		0.72	1	0.395	
Initial mass		1.69	1	0.193	
Mass gain 6-24h post-challenge		0.08	1	0.781	0.782
**Treatment**		10.7	1	**0.001**	**0.003**
**OXY**					
Brood size	0.59		1, 28	0.450	
Date	0.18		1, 30	0.678	
Sex	3.15		1, 69	0.081	
Initial mass	6.10		1, 79	0.016	
**Mass gain 6-24h post-challenge**	12.0		1, 87	**0.001**	**0.003**
**Treatment**	9.83		1, 64	**0.003**	**0.004**

Journal of Experimental Biology • Supplementary information

Table S6. Effect of experimental treatment (LPS or Control) on MDA levels and the probability of showing detectable levels of ROMs in plasma at 24h post-treatment, considering initial pre-treatment values and incorporating plasma triglyceride levels as covariate in both models. We show F and df, and P-values for all fixed effects. Significant *P*-value for the treatment effect are in bold. Initial MDA and ROM levels are computed as residuals of each variable after controlling for triglyceride levels at that sampling time.

	F	χ2	df	*P*	Corrected *P*
**MDA**					
Brood size	0.12		1, 30	0.732	
Date	0.04		1, 29	0.852	
Sex	0.18		1, 80	0.669	
Initial mass	3.73		1, 87	0.057	
Initial MDA (residuals)	64.0		1, 50	<0.001	
Triglyceride levels	8.87		1, 68	0.004	
Treatment	0.15		1, 65	0.696	0.696
**ROMs (prob)**					
Brood size	0.46	1		0.499	
Date	<0.01	1		0.964	
Sex	0.53	1		0.465	
Initial mass	2.31	1		0.128	
Initial ROMs (prob) (residuals)	0.41	1		0.52	
Triglyceride levels	0.06	1		0.805	
**Treatment**	34.0	1		**<0.001**	**<0.001**

Journal of Experimental Biology • Supplementary information
